# Hamiltonian decompositions of 4‐regular Cayley graphs of infinite abelian groups

**DOI:** 10.1002/jgt.22840

**Published:** 2022-05-09

**Authors:** Joshua Erde, Florian Lehner

**Affiliations:** ^1^ Institute of Discrete Mathematics Graz University of Technology Graz Austria

**Keywords:** alspach's conjecture, cayley graph, Hamiltonian cycle, Hamiltonian decomposition

## Abstract

A well‐known conjecture of Alspach says that every 2k‐regular Cayley graph of a finite abelian group can be decomposed into Hamiltonian cycles. We consider an analogous question for infinite abelian groups. In this setting one natural analogue of a Hamiltonian cycle is a spanning double‐ray. However, a naive generalisation of Alspach's conjecture fails to hold in this setting due to the existence of 2k‐regular Cayley graphs with finite cuts F, where ∣F∣ and k differ in parity, which necessarily preclude the existence of a decomposition into spanning double‐rays. We show that every 4‐regular Cayley graph of an infinite abelian group all of whose finite cuts are even can be decomposed into spanning double‐rays, and so characterise when such decompositions exist. We also characterise when such graphs can be decomposed either into Hamiltonian circles, a more topological generalisation of a Hamiltonian cycle in infinite graphs, or into a Hamiltonian circle and a spanning double‐ray.

## 1 INTRODUCTION

A *Hamiltonian cycle* in a finite graph G is a cycle which includes every vertex of the graph; a *Hamiltonian decomposition* is a partition of the edge set of G into disjoint sets $E=E_{1}\;⊎\;E_{2}\;⊎\;\;\text{⋯}\;\;⊎\;E_{r}$, where each $E_{i}$ is a Hamiltonian cycle in G. One of the earliest results in graph theory is a theorem of Walecki from 1890 stating that every finite complete graph of odd order has a Hamiltonian decomposition (see, e.g., [3]). Since then, Hamiltonian decompositions of various classes of graphs have been studied, the survey of Alspach et al. [4] gives an overview.

Given an abelian group (Γ,+) and a generating set S⊆Γ, the *Cayley graph of* Γ *with respect to* S, which we denote by Cay(Γ,S), is the simple graph whose vertex set is Γ and in which the neighbours of a vertex x are given by the vertices {x+s:s∈S}. It is a folklore result that every connected Cayley graph of a finite abelian group contains a Hamiltonian cycle, hence it is natural to ask for which of them we can find a Hamiltonian decomposition. Sometimes this fails for parity reasons. Indeed, if a graph has a Hamiltonian decomposition then it must be 2k‐regular for some k. It is a long‐standing conjecture of Alspach that for Cayley graphs of finite abelian groups this is the only thing that can go wrong.

Conjecture 1 ((Alspach [1, 2])) If Γ is a finite abelian group and S generates Γ, then the Cayley graph Cay(Γ,S) has a Hamiltonian decomposition, provided that it is 2k‐regular for some k.

Not much is known about this conjecture. If k=1, then it trivially holds, and in case k=2 it was proved by Bermond et al. [5]. However, even the case k=3 is still open, although partial results towards this case were given by Liu [15], Westlund [19] and Dean [7]. Liu also showed [16, 17] that the conjecture holds for any k when S is a minimal generating set.

While the previous results all concerned finite graphs, Hamiltonian cycles have also been considered in infinite graphs. It is not immediate what the correct generalisation of a Hamiltonian cycle to an infinite graph should be. One natural structure to consider is a spanning *double‐ray*, an infinite connected graph in which each vertex has degree two, which we will refer to as a *Hamiltonian double‐ray*.

Nash‐Williams [18] showed that every connected Cayley graph of a finitely generated infinite abelian group contains a Hamiltonian double‐ray, and together with a result of Witte [20], this then implies that every connected Cayley graph of a finitely generated infinite abelian group with infinite degree (i.e., S is infinite) has a decomposition into Hamiltonian double‐rays. More recently, the authors and Pitz [13] showed that if Γ is a finitely generated abelian group, every element of the finite generating set S has infinite order, and Cay(Γ,S) is one‐ended, then it has a decomposition into Hamiltonian double‐rays. Bryant et al. [6] considered the specific case Γ=Z and gave conditions on S which guarantee the existence of a decomposition of Cay(Z,S) into Hamiltonian double‐rays.

Besides G having to be 2k‐regular for some k, there is another parity obstruction to the existence of a decomposition into Hamiltonian double‐rays. A *cut* is a partition of the vertex set into two parts called the *sides* of the cut; it is called finite if there are finitely many edges connecting the two sides, called *cross edges* or simply *edges* of the cut. If F is a finite cut both of whose sides are infinite, then any Hamiltonian double‐ray must contain an odd number of edges of F, otherwise it only contains finitely many vertices on one of the sides. So a decomposition into Hamiltonian double‐rays can only exist if the number of cross edges of F has the same parity as k. Note that, when G is one‐ended this parity condition does not play a role since there are no finite cuts with two infinite sides.

In this paper, we restrict our attention to 4‐regular Cayley graphs of infinite abelian groups. For such graphs, we can assume that either $\Gamma =Z^{2}$, or Γ=Z, or $\Gamma =Z⊕Z_{i}$ for some i, see Proposition 9 and the discussion thereafter. Moreover, for such Cayley graphs the parity condition on finite cuts mentioned above boils down to the following:

(P) Every finite cut contains an even number of edges.

The existence of a decomposition into Hamiltonian double‐rays in the case $\Gamma =Z^{2}$ follows from the work of the authors and Pitz in [13], also see [14, proposition 5] for a short, direct proof. Bryant et al. [6] considered the case Γ=Z and showed among other things that if S is any generating set with ∣S∣=2, then Cay(Z,S) has a decomposition into Hamiltonian double‐rays if it satisfies (P). In this paper we extend this result to groups of the form $\Gamma =Z⊕Z_{i}$ for some i, thus proving the following result.

Theorem 2 Let G be a connected, 4‐regular Cayley graph of an infinite abelian group which satisfies (P), then G has a decomposition into Hamiltonian double‐rays.

Our proof also gives Hamiltonian decompositions for a different notion of infinite Hamiltonian cycles called *Hamiltonian circles*. The notion is based on a topological approach to infinite graph theory, a comprehensive introduction to which can be found in [8, 9, 11]. We defer the precise definitions to Section 2.1, but mention that any Hamiltonian circle meets any finite cut in an even number of edges (see Lemma 5). Thus (P) is also necessary for a decomposition of a 4‐regular graph into Hamiltonian circles to exist. Once again, it turns out that for 4‐regular Cayley graphs of abelian groups, (P) is also sufficient.

Theorem 3 Let G be a connected, 4‐regular Cayley graph of an infinite abelian group which satisfies (P), then G has a decomposition into Hamiltonian circles.

Finally, in the case that (P) does not hold, we are able to find a ‘mixed’ decomposition into a Hamiltonian double‐ray and a Hamiltonian circle.

Theorem 4 Let G be a connected, 4‐regular Cayley graph of an infinite abelian group which does not satisfy (P), then G has a decomposition into a Hamiltonian double‐ray and a Hamiltonian circle.

## 2 PRELIMINARIES

### 2.1 Topological infinite graph theory

A graph G is *locally finite* if every vertex has finite degree. A *ray* in a graph is a one‐way infinite path, and an *end* of a locally finite graph is an equivalence class of rays under the relation $R_{1}\sim R_{2}$ if for every finite cut F, all but finitely many vertices of $R_{1}$ and $R_{2}$ lie on the same side of F. We note that if Γ is a finitely generated group then the number of ends of Cay(Γ,S) does not depend on the choice of S, see for example [12].

If we denote by Ω the set of ends of a graph G then there is a natural topology on the 1‐complex of G together with Ω which forms a compact topological space known as the *Freudenthal compactification of* G which is normally denoted by ∣G∣. A *circle* in G is a subspace of ∣G∣ homeomorphic to the circle $S_{1}$. It can be shown that a circle is uniquely defined by the set of edges contained in it, so by a slight abuse of notation we will also call this set of edges a circle.

It is worth noting that there is an equivalent, combinatorial definition of a circle, generalising the fact that a cycle is an inclusion minimal element of the cycle space of a graph.

Lemma 5 ((Diestel and Kühn [10])) Let G be a locally finite graph. Then a set of edges C is a circle if and only if C meets every finite cut F of G in an even number of edges, and there is no nonempty $C^{\prime }\;⊊\;C$ with this property.

A *Hamiltonian circle* is a circle which meets every vertex of G. It is relatively easy to show that every Hamiltonian circle in a one‐ended graph is a spanning double‐ray. For two‐ended graphs it can be shown that every Hamiltonian circle is a disjoint union of two double‐rays which together span G, each of which is a union of two rays, one going to each end of the graph. However, for our purposes we will only need the converse of both of these statements, that such a subgraph is a Hamiltonian circle, which is a simple consequence of Lemma 5 and whose proof we provide for completeness.

Lemma 6

1. If G is a locally finite, one‐ended graph and C is a spanning double‐ray, then C is a Hamiltonian circle.
2. If G is a locally finite, two‐ended graph and C is a disjoint union of two double‐rays which together span G, each of which contains a ray to both ends of the graph, then C is a Hamiltonian circle.

In the case that G is one‐ended, every finite cut F has a unique infinite component, which must contain both tails of C, and hence C must meet F in an even number edges. For every nonempty strict subset C′ of C, there is at least one vertex only incident to one edge in C′. Thus C′ meets the cut with this vertex on one side and all other vertices on the other side in only one edge. It follows from Lemma 5 that C is a Hamiltonian circle.

In the case that G is two‐ended, every finite cut F has either one, or two infinite components. Let $C_{1}$ and $C_{2}$ be the two double‐rays forming C. If F has one infinite component, then both tails of $C_{1}$ and $C_{2}$ are contained in this component, and so both must meet F in an even number of edges. In the second case the two tails of $C_{1}$ and $C_{2}$ are contained in different components, and so both must meet F in an odd number of edges. In either case, C meets F in an even number of edges. If C′⊊C is nonempty, then either there is a vertex incident to only one edge in C′, or C′ is one of $C_{1}$ and $C_{2}$. In the first case we can use the same argument as above to show that C′ meets some finite cut in an odd number of edges. Otherwise, let F be a finite cut which separates the tails of two rays contained in C′. Then C′ must contain infinitely many vertices on both sides of F and thus it contains an odd number of cross edges of F. Consequently, by Lemma 5, C is a Hamiltonian circle.  □

### 2.2 Structure of 4‐regular Cayley graphs of abelian groups

It will be useful to give a classification of the possible graphs that can arise as 4‐regular Cayley graphs of infinite abelian groups.

Definition 7 For any k∈N and l∈Z, the graph $G_{k,l}$ is the graph with

$$V\left(G_{k,l}\right)=\left\{\left(m,n\right)|m,n\in Z,0\leq m<k\right\},$$

and whose edge set consists of the following three kinds of edges:

(a) (m,n) to (m,n+1) for m,n∈Z,0≤m<k,
(b) (m,n) to (m+1,n) for m,n∈Z,0≤m<k−1, and
(c) (k−1,n) to (0,n−l) for n∈Z.

**Figure 1:**
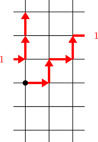
The walk P in $G_{3,1}$ starting at the black dot at (0,0). The label indicates where the edge from (2,2) to (0,1) leaves and enters the diagram. [Color figure can be viewed at wileyonlinelibrary.com]

In all figures throughout this paper, we represent $G_{k,l}$ as follows. We draw every vertex (m,n) at coordinates (m,n) in the plane. Edges of type (a) and (b) are drawn as straight line segments, edges of type (c) are represented by two half edges to the right of (k−1,n) and to the left of (0,n−l). Numbers next to these half edges indicate which of them correspond to the same edge, see for instance Figure 1. We will refer to edges of type (a) as *vertical* edges and to edges of types (b) and (c) as *horizontal* edges.

The aim of this section is to show that any 4‐regular Cayley graph of an abelian group apart from the square grid is in fact of the form $G_{k,l}$ for some k and l. To this end, the following observation will be useful.

Observation 8 Let Γ,Δ be isomorphic groups. Then every group isomorphism ϕ:Γ→Δ is also a graph isomorphism $\text{Cay}\left(\Gamma ,\left\{s_{1},\text{…},s_{k}\right\}\right)\to \text{Cay}\left(\Delta ,\left\{\phi \left(s_{1}\right),\text{…},\phi \left(s_{k}\right)\right\}\right)$. This also holds for *epimorphisms*, that is, surjective homomorphisms.

Proposition 9 If G is a connected 4‐regular Cayley graph of an infinite abelian group then either G is the square grid, or there exists k∈N and l∈Z such that $G≃G_{k,l}$.

If G=Cay(Γ,S) is a connected 4‐regular Cayley graph of an abelian group, then 2≤∣S∣≤4. If ∣S∣=4, then all of the generators must be involutions and the group is finite. If ∣S∣=3, then two of the generators are involutions and hence the group is either finite (in case the third generator has finite order) or $\Gamma =Z⊕Z_{2}⊕Z_{2}=Z⊕K_{4}$, where $K_{4}$ is the Klein four‐group. In the latter case it is easy to verify that $G=G_{4,0}$ is the only possibility if two involutions of $K_{4}$ appear in the generating set.

So assume that S={a,b}. In this case there is a unique epimorphism $\phi :Z^{2}\to \Gamma$ which maps (1,0) to a and (0,1) to b. By the isomorphism theorem, $\Gamma ≃Z^{2}∕\text{ker}\phi$ and by Observation 8 the respective Cayley graphs are isomorphic as well. So it suffices to study Cayley graphs of groups of the form $\Gamma =Z^{2}∕N$ with generators (1,0) and (0,1), where N is any subgroup of $Z^{2}$.

If $N≃Z^{2}$ then $Z^{2}∕N$ is finite. If N={(0,0)}, then $\Gamma =Z^{2}$ and the Cayley graph is the square grid. The only remaining case is when N is infinite cyclic, that is, there are k,l∈Z such that N={n⋅(k,l)∣n∈Z}. We can without loss of generality assume that k>0—clearly k and l cannot simultaneously be 0 and exchanging their roles leads to an isomorphic situation. Furthermore, if necessary we can replace (k,l) by (−k,−l). Now looking at {(m,n)∣m,n∈Z,0≤m<k} as a system of representatives it is straightforward to check that the resulting graph is isomorphic to $G_{k,l}$. □

Note that every $G_{k,l}$ occurs as a Cayley graph, more precisely, it is the Cayley graph of the group $\Gamma_{k,l}≔Z⊕Z_{\text{gcd}\left(k,l\right)}$ with generators 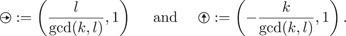


For gcd(k,l)=1 we note that $Z⊕Z_{1}=Z$ and consistently with the above, $G_{k,l}$ is the Cayley graph of the group $\Gamma_{k,l}=Z$ with generators 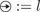 and 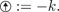.

Using this representation of $G_{k,l}$, vertical edges correspond to the generator 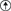 and horizontal edges correspond to the generator 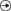. For 0≤m<k the vertex (m,n) of $G_{k,l}$ corresponds to the group element 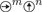; we point out that if we refer to a vertex of $G_{k,l}$ or group element of $\Gamma_{k,l}$ as a pair (m,n), we always interpret it as 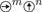, and never as $\left(m\in Z,n\in Z_{\text{gcd}\left(k,l\right)}\right)\in Z\times Z_{\text{gcd}\left(k,l\right)}$.

Note that we do not need to consider the group $Z⊕V_{4}$ since $G_{4,0}$ also occurs as a Cayley graph of $\Gamma_{4,0}=Z⊕Z_{4}$. Further note that (by replacing generators by their inverses and swapping their roles) we have that $G_{k,l}≃G_{k,-l}$, $G_{k,l}≃G_{l,k}$ for l>0, and $G_{k,l}≃G_{-l,-k}$ for l<0.

We can specify a walk in $G_{k,l}$ by giving the starting vertex together with a series of generators and their inverses, denoted by 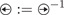 and 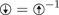. To avoid confusion with the group element obtained by multiplication of these generators, we will put the generators defining the walk in square brackets. For a more compact representation we will also represent repeated patterns by exponentiation. For example, the following expressions all define the same walk in $G_{3,1}$, see Figure 1: 
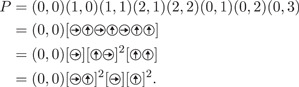


## 3 HAMILTONIAN DECOMPOSITIONS OF 4‐REGULAR CAYLEY GRAPHS

A *vertical cut* of $G_{k,l}$ is the orbit of a horizontal edge under the action of the subgroup generated by 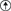, or in other words, the set all vertical translates of a horizontal edge. A *horizontal cut* of $G_{k,l}$ is the orbit of a vertical edge under the action of the subgroup generated by 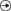. Let $\tilde{E}$ be a subset of the edges of $G_{k,l}$. We say that $\tilde{E}$ *prevails* in a vertical (horizontal) cut, if for every edge e in this cut there are a>0 and b>0 such that 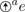 and 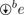 (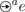 and 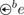) lie in $\tilde{E}$. We say that $\tilde{E}$ is *horizontally (vertically) prevalent* if there is a horizontal (vertical) cut in which $\tilde{E}$ prevails and *bi‐prevalent* if it is both horizontally and vertically prevalent. We say that a decomposition $E_{1}⊎E_{2}$ is *horizontally prevalent* if both $E_{1}$ and $E_{2}$ are horizontally prevalent, and similarly for vertically and bi‐prevalent.

Lemma 10

1. If $G_{k,l}$ admits a vertically prevalent or bi‐prevalent decomposition into Hamiltonian double‐rays, then so does $G_{k+2,l}$.
2. If l>0 and $G_{k,l}$ admits a horizontally prevalent or bi‐prevalent decomposition into Hamiltonian double‐rays, then so does $G_{k,l+2}$.
3. Analogous statements hold for decompositions into Hamiltonian circles, and for decompositions into a Hamiltonian double‐ray and a Hamiltonian circle.

For the proof of the first statement let $E_{1}⊎E_{2}$ be a vertically prevalent decomposition of $G_{k,l}$ into Hamiltonian double‐rays, and let C be a vertical cut in which both $E_{1}$ and $E_{2}$ prevail. Without loss of generality, C consists of all edges connecting (k−1,n) to (0,n−l) for n∈Z; this can always be achieved by applying an appropriate automorphism. We write $e_{n}$ for the edge connecting (k−1,n) to (0,n−l). If $e_{n}\in E_{1}$, then we define $h_{n}=\min\left\{h>0|e_{n+h}\in E_{1}\right\}$. Similarly, if $e_{n}\in E_{2}$, then we define $h_{n}=\min\left\{h>0|e_{n+h}\in E_{2}\right\}$.

**Figure 2:**
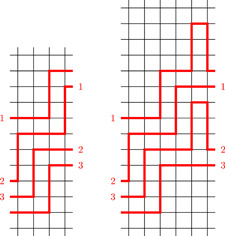
Extending a bi‐prevalent decomposition of $G_{4,2}$ into Hamiltonian double‐rays to a bi‐prevalent decomposition of $G_{6,2}$. [Color figure can be viewed at wileyonlinelibrary.com]

Next note that $G_{k+2,l}$ can be obtained from $G_{k,l}$ by the following procedure: remove all edges in C, and for every j∈Z add vertices (k,j) and (k+1,j) and the appropriate edges. Using this construction of $G_{k+2,l}$ we transform $E_{1}$ and $E_{2}$ into subsets $E_{1}^{\prime }$ and $E_{2}^{\prime }$ of the edge set of $G_{k+2,l}$ as follows. For i∈{1,2}, the set $E_{i}^{\prime }$ consists of $E_{i}⧹C$ and the edges of the walks 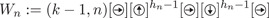 for every n with $e_{n}\in E_{i}$. See Figure 2 for an example in the case of $G_{4,2}$.

Vertical prevalence of $E_{1}$ and $E_{2}$ ensures that $h_{n}$ is finite, thus $W_{n}$ is a finite walk. Note that $W_{n}$ starts at (k−1,n), ends at (0,n−l), and additionally contains the vertices (k,j) and (k+1,j) for $n\leq j<n+h_{n}$. In particular, by definition of $h_{n}$, the paths in $\left\{W_{n}|e_{n}\in E_{i}\cap C\right\}$ are vertex disjoint and their union covers the vertices (k,j) and (k+1,j) for j∈Z.

We now show that $E_{1}^{\prime }$ and $E_{2}^{\prime }$ form the desired decomposition of $G_{k+2,l}$. The graph spanned by $E_{i}^{\prime }$ is obtained from the graph spanned by $E_{i}$ by replacing edges in C by disjoint paths with the same endpoints. Since the graph spanned by $E_{i}$ was connected and 2‐regular, the same is true for the graph spanned by $E_{i}^{\prime }$. It contains all vertices (i,j) for i<k and j∈Z since $E_{i}$ was spanning, and it contains all vertices (k,j) and (k+1,j) due to the above observation.

To see that $E_{1}^{\prime }$ and $E_{2}^{\prime }$ are disjoint, first note that $E_{1}⧹C$ and $E_{2}⧹C$ are disjoint, so we only need to show that the walks $W_{n}$ are edge disjoint. Take $e_{n}\in E_{1}$ and $e_{m}\in E_{2}$. If $W_{n}$ and $W_{m}$ intersect in a horizontal edge, then either n=m (for the first and last edge), or $n+h_{n}=m+h_{m}$ (for the central edge). This is not possible because $e_{n}$ and $e_{n+h_{n}}$ are in $E_{1}$ whereas $e_{m}$ and $e_{m+h_{m}}$ are in $E_{2}$. For vertical edges, note that if $W_{n}$ contains an edge from (k,j) to (k,j+1) or from (k+1,j+1) to (k+1,j), then $e_{j+1}\in E_{2}$. Similarly, if $W_{m}$ contains such an edge, then $e_{j+1}\in E_{1}$. This implies that they cannot contain the same vertical edge, so $W_{n}$ and $W_{m}$ must be disjoint.

The decomposition $E_{1}^{\prime }\;⊎\;E_{2}^{\prime }$ is vertically prevalent since $E_{i}^{\prime }$ contains the edge from (k+1,n) to (0,n−l) if and only if $E_{i}$ contains an edge from (k−1,n) to (0,n−l). If we additionally assume that $E_{1}\;⊎\;E_{2}$ is horizontally prevalent, then so is $E_{1}^{\prime }\;⊎\;E_{2}^{\prime }$ since any horizontal cut in $G_{k,l}$ is fully contained in a horizontal cut in $G_{k+2,l}$.

This finishes the proof of the first statement. The second statement follows from the fact that for l>0 there is an isomorphism between $G_{k,l}$ and $G_{l,k}$ which swaps horizontal and vertical cuts. The third statement can be proved in a completely analogous fashion (with the additional observation that $E_{i}$ having tails in different ends of $G_{k,l}$ implies that $E_{i}^{\prime }$ has tails in different ends of $G_{k+2,l}$), we leave the details to the reader.  □

Lemma 11 Let Γ be a 2‐ended abelian group, let S be a generating set of Γ, and let Δ be an infinite cyclic subgroup of Γ generated by a. Let G be the Cayley graph of Γ with respect to S and let H be the Cayley graph of Γ∕Δ with respect to the generating set SΔ, where we allow multiple edges in case $s_{1}\Delta =s_{2}\Delta$ for $s_{1},s_{2}\in S$, and let π:G→H be the projection map. Let C be a Hamiltonian cycle in H and let k be the sum of the generators used along this cycle.

1. If k=a, then $\pi^{-1}\left(C\right)$ is a Hamiltonian double‐ray in G.
2. If $k=a^{2}$, then $\pi^{-1}\left(C\right)$ is a Hamiltonian circle in G.

Every vertex v of G has exactly two incident edges in the preimage of C (namely the two edges corresponding to the same generators as the edges in C incident to π(v)). Hence $\pi^{-1}\left(C\right)$ is 2‐regular.

Since C is spanning in H we know that every component of $\pi^{-1}\left(C\right)$ contains elements of all cosets with respect to Δ. Moreover, if k=a, then by following the edges corresponding to the same generators as edges along C, we see that for any element v of Γ the element va lies in the same component of $\pi^{-1}\left(C\right)$ as v. Hence in this case $\pi^{-1}\left(C\right)$ is connected and thus a Hamiltonian double‐ray.

If $k=a^{2}$, then a similar argument shows that v and $va^{i}$ lie in the same component if and only if i is even, so $\pi^{-1}\left(C\right)$ has exactly two components. Each of the components is invariant under the action of $a^{2}$ and since high positive and negative powers of a converge to different ends of G we conclude that each component contains tails in both ends. Thus $\pi^{-1}\left(C\right)$ is a Hamiltonian circle.   □

Lemma 12

1. If 3≤k=l+2, then $G_{k,l}$ has a bi‐prevalent decomposition into Hamiltonian double‐rays.
2. If 3≤k=l+3, then $G_{k,l}$ has a bi‐prevalent decomposition into one Hamiltonian double‐ray and one Hamiltonian circle.
3. If 3≤k=l+4, then $G_{k,l}$ has a bi‐prevalent decomposition into Hamiltonian circles.

Let A be the group generated by 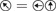. Note that the quotient group $\Gamma_{k,l}∕A$ is a cyclic group generated by 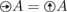. The Cayley graph H of this quotient group is a cycle where each edge has been replaced by two parallel edges (corresponding to the two different generators).

**Figure 3:**
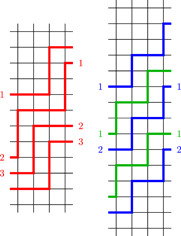
A bi‐prevalent double‐ray in $G_{4,2}$ whose complement is a bi‐prevalent double‐ray and a bi‐prevalent circle in $G_{4,0}$ whose complement is a bi‐prevalent circle. [Color figure can be viewed at wileyonlinelibrary.com]

If k=l+2, pick a Hamiltonian cycle C of H using exactly k−1 edges corresponding to the generator 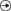. Recall that 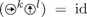, so C contains l+1=k−1 edges corresponding to the generator 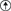 and the same is true for the (edge‐)complement of C. Note that 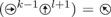. Thus Lemma 11 implies that the preimages $E_{1}$ of C and $E_{2}$ of its complement under the natural projection map form a decomposition of $G_{k,l}$ into Hamiltonian double‐rays. See the left picture in Figure 3 for an example in $G_{4,2}$.

The argument for the case k=l+4 is completely analogous, but with k−2=l+2 edges corresponding to generators 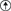 and 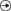, respectively; note that 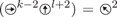. See the right picture in Figure 3 for an example in $G_{4,0}$.

**Figure 4:**
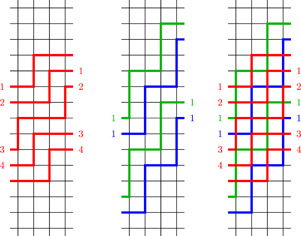
A bi‐prevalent double‐ray in $G_{4,1}$ whose complement is a bi‐prevalent circle. [Color figure can be viewed at wileyonlinelibrary.com]

For k=l+3 we choose the cycle C with k−1 edges corresponding to the generator 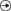 and (consequently) l+1=k−2 edges corresponding to the generator 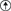. Thus the complement will contain k−2 edges corresponding to the generator 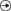 and l+2 edges corresponding to the generator 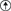. It follows that the preimage $E_{1}$ of C is a Hamiltonian double‐ray and the preimage $E_{2}$ of its complement is a Hamiltonian circle. See Figure 4 for an example in $G_{4,1}$.

It remains to show that the decompositions are bi‐prevalent. If we follow the edges of $E_{1}$ starting at id then the first edge corresponding to 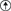 lies in the same horizontal cut H as the edge from id to 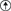, and the first edge corresponding to 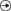 lies in the same vertical cut K as the edge from id to 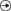. Since C contains edges of both types, we know that $E_{1}$ contains at least one edge in both of these cuts. Similarly, $E_{2}$ contains edges in both of these cuts.

Note that 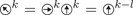. Since the decomposition $E_{1}\;⊎\;E_{2}$ is invariant under the action of A, this implies that for every edge $e\in E_{i}\cap K$ the edges 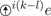 for i∈Z are also in $E_{i}\cap K$; thus the decomposition is vertically prevalent. If l=0, then the horizontal cut H is finite, and the decomposition is horizontally prevalent because both parts intersect with H, otherwise the same argument as for K applies.   □

Note that the restriction k≥3 in the first condition is necessary since $G_{1,-1}$ and $G_{2,0}$ are not 4‐regular. In the other two cases it is merely required to enable us to apply Lemma 11; if k was smaller than 3 in these cases then the quotient group would become trivial or infinite.

By Proposition 9 the only 4‐regular Cayley graphs of abelian groups are either the square grid, or of the form $G_{k,l}$. Since the square grid satisfies the conclusion of Theorem 2, it will suffice to show that every 4‐regular $G_{k,l}$ which satisfies (P) has a decomposition into Hamiltonian double‐rays and a decomposition into Hamiltonian circles. It is easy to check that (P) is satisfied if and only if k and l have the same parity. Hence the following lemma completes the proof of Theorems 2, 3 and 4.

Lemma 13 Let k∈N and l∈Z be such that $G_{k,l}$ is 4‐regular.

1. If k≡lmod2, then $G_{k,l}$ has a decomposition into two Hamiltonian double‐rays.
2. If k≡lmod2, then $G_{k,l}$ has a decomposition into two Hamiltonian circles.
3. If k≢lmod2, then $G_{k,l}$ has a decomposition into a Hamiltonian double‐ray and a Hamiltonian circle.

By the remark after Proposition 9 it is sufficient to prove the lemma for k≥l≥0. Moreover, we can ignore the cases k=1,l≤1 and k=2,l=0 since they do not lead to 4‐regular graphs.

**Figure 5:**
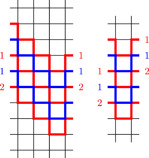
A bi‐prevalent decomposition of $G_{4,0}$ and a vertically prevalent decomposition of $G_{2,2}$ into Hamiltonian double‐rays. [Color figure can be viewed at wileyonlinelibrary.com]

For the first part, note that by Lemma 12, there are bi‐prevalent decompositions of $G_{4,2}$ and $G_{3,1}$ into Hamiltonian double‐rays. Lemma 10 and induction finish the proof apart from the cases k=l=2 and k>l=0. For l=0 it is enough to show that $G_{4,0}$ has a vertically prevalent decomposition into Hamiltonian double‐rays, once this is done we can induct using the first part of Lemma 10. For k=l=2 we explicitly construct a decomposition. Both of these are presented in Figure 5.

**Figure 6:**
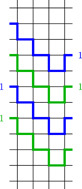
A bi‐prevalent decomposition of $G_{4,2}$ into Hamiltonian circles (note that the complement of this circle is a vertical translation of it). [Color figure can be viewed at wileyonlinelibrary.com]

To prove the second statement, recall that there is an isomorphism between $G_{k,l}$ and $G_{k,-l}$ and note that this isomorphism preserves horizontal and vertical cuts. Hence by Lemma 12 the graphs $G_{4,0}$ and $G_{3,1}≃G_{3,-1}$ have bi‐prevalent decompositions into Hamiltonian circles. Furthermore we give in Figure 6 a bi‐prevalent decomposition of $G_{4,2}$ into Hamiltonian circles. An inductive application of Lemma 10 then finishes the proof of the second part apart from the case k=l=2. To see that $G_{2,2}$ has a decomposition into Hamiltonian circles, simply consider the decomposition into horizontal and vertical edges.

For the proof of the third part note that by Lemma 12 the graphs $G_{5,2}$, $G_{4,1}$, and $G_{3,0}$ have bi‐prevalent decompositions into a Hamiltonian double‐ray and a Hamiltonian circle. Inductive application of Lemma 10 finishes the proof apart from the case k=2,l=1. However, the graph $G_{2,1}$ has a decomposition into a Hamiltonian double‐ray and a Hamiltonian circle as well; again consider the decomposition into vertical and horizontal edges.  □

## 4 A GENERALISATION OF ALSPACH'S CONJECTURE

The conditions in Alspach's conjecture arise quite naturally; since every Hamiltonian cycle must meet every cut of a graph in an even number of edges, for a Hamiltonian decomposition to exist each cut must be even, and this is equivalent in a finite graph to insisting that each vertex has even degree.

If we consider Hamiltonian circles, which again meet every finite cut of an infinite graph in an even number of edges, then clearly (P) is again necessary for a decomposition into Hamiltonian circles to exist, and a natural generalisation of Alspach's conjecture would be that (P) is also sufficient. Theorem 3 shows that this is true for 4‐regular Cayley graphs.

A Hamiltonian double‐ray, however, meets a finite cut of an infinite graph an even number of times if the cut has one infinite component, and an odd number of times if it has two infinite components. Hence in order for a decomposition into k Hamiltonian double‐rays to exist the number of edges in every finite cut with one infinite component must be even, and the number of edges in every finite cut with two infinite components must have the same parity as k. A simple double counting argument shows that the first condition will always hold if G is 2k‐regular, and so perhaps a natural generalisation of Alspach's conjecture for double‐rays would be that, together with 2k‐regularity, this second condition is also sufficient. Again Theorem 2 shows that this is true for 4‐regular Cayley graphs.

In light of Theorem 4, an even more ambitious conjecture, would be the following. Let

(Q*i*) Every finite cut F with two infinite components satisfies ∣F∣≡imod2.

Conjecture 14 Let G be a 2k‐regular Cayley graph of an abelian group. If G satisfies (Q*i*) then G has a decomposition into i Hamiltonian double‐rays and k−i Hamiltonian circles.
